# The Editor’s Fever Reports

**Published:** 1851-10

**Authors:** 


*The Editor's Fever Reports.*— We must solicit indulgence toward our protracted articles on Continued Fever. We are fully aware that, appearing as they do in broken portions, by many of our readers they will not be considered very interesting, and perhaps scarcely readable. We trust that, when completed, they will be thought to possess some value for reference. We would add, by way of furthering our petition for patience, that the subjects which will come up after the statistical investigations are completed, viz., the *diagnosis*, *identity of Typhus and Typhoid*, and the *treatment*, may, possibly, be more acceptable than the numerical details which will occupy us for the two or three succeeding numbers. To the obdurate critic we can only say, 'pray give the articles a quiet go-by, and enjoy the complacent satisfaction of being able to dispose of an annoyance so easily and effectually!'

